# Supplementary figures and images for: The influence of body composition and fat distribution on circadian blood pressure rhythm and nocturnal mean arterial pressure dipping in patients with obesity
Source: PLoS One. 2023 Jan 31;18(1):e0281151. doi: 10.1371/journal.pone.0281151 (PMC9888712; doi:10.1371/journal.pone.0281151)

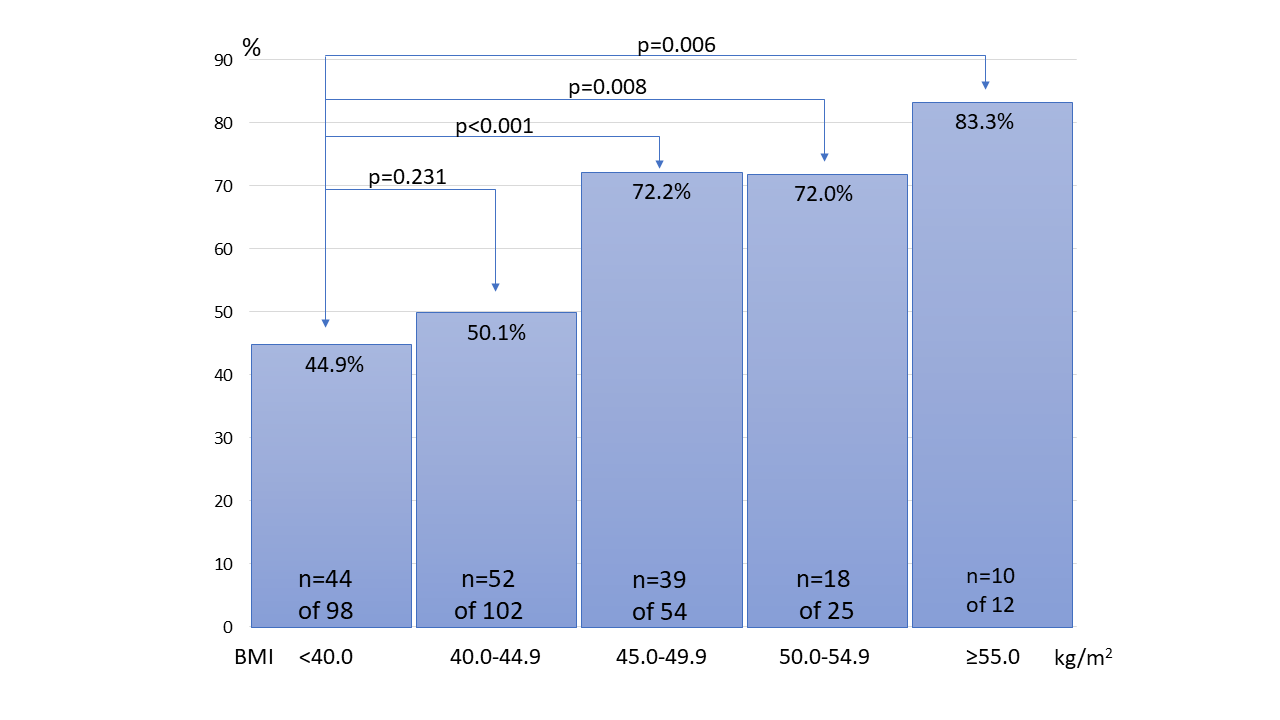

Supplement: S1 Fig — (TIF) [file pone.0281151.s001.tif]

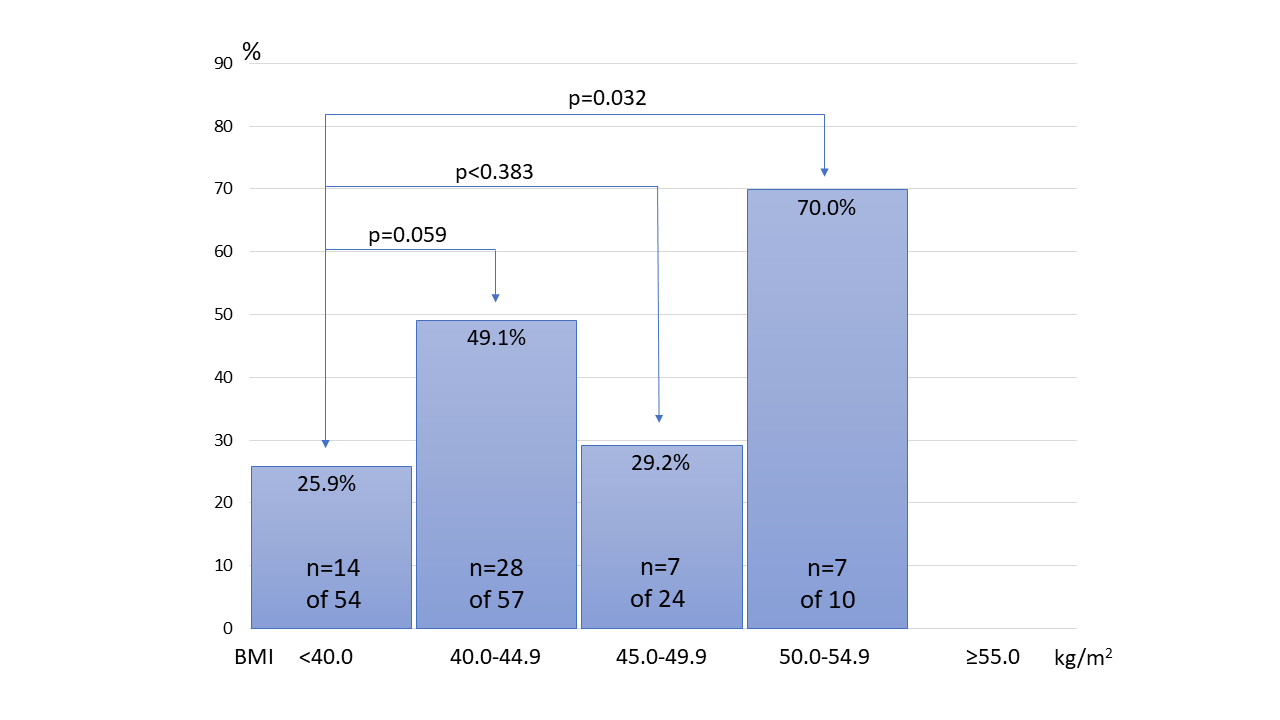

Supplement: S2 Fig — (TIF) [file pone.0281151.s002.tif]
