# Supplementary material for: The influence of body composition and fat distribution on circadian blood pressure rhythm and nocturnal mean arterial pressure dipping in patients with obesity
Source: PLoS One. 2023 Jan 31;18(1):e0281151. doi: 10.1371/journal.pone.0281151 (PMC9888712; doi:10.1371/journal.pone.0281151)
Supplement: S1 Table — (DOCX) [file pone.0281151.s003.docx]

S 1 Table. The number and percentage (in brackets) of hypertensive and normotensive women and men

|  | Hypertensive | Normotensive | p-value |
| --- | --- | --- | --- |
| Women | 179 (41.1) | 127 (29.1) | p = 0.033 |
| Men | 112 (25.7) | 18 (4.1) | p < 0.001 |
| p-value | p = 0.005 | p < 0.001 |  |
