# Supplementary material for: The influence of body composition and fat distribution on circadian blood pressure rhythm and nocturnal mean arterial pressure dipping in patients with obesity
Source: PLoS One. 2023 Jan 31;18(1):e0281151. doi: 10.1371/journal.pone.0281151 (PMC9888712; doi:10.1371/journal.pone.0281151)
Supplement: S2 Table — (DOCX) [file pone.0281151.s004.docx]

S 2 Table. Anthropometric parameters and body composition in hypertensive and normotensive women with obesity

|  | Hypertensive women (n=179) | Normotensive women (n=127) | p-value |
| --- | --- | --- | --- |
| BW (kg) | 114.7 ± 18.5 | 115.9 ± 15.4 | 0.578 |
| BMI (kg/m^2^) | 42.9 ± 6.2 | 41.9 ± 5.0 | 0.126 |
| WC (cm) | 121 ± 12 | 119 ± 12 | 0.210 |
| HC (cm) | 131 ± 16 | 130 ± 12 | 0.831 |
| WHR (cm/cm) | 0.94 ± 0.09 | 0.92 ± 0.08 | 0.155 |
| LBM (kg) | 57.4 ± 7.7 | 58.4 ± 6.6 | 0.293 |
| FM (kg) | 49.3 ± 10.4 | 51.9 ± 9.8 | 0.042 |
| BF% | 44.4 ± 4.3 | 45.7 ± 3.7 | 0.101 |
| PerF (kg) | 35.5 ± 8.9 | 39.2 ± 8.6 | 0.019 |
| AbdF (kg) | 12.7 ± 2.3 | 12.7 ± 2.3 | 0.999 |
| AbdF/FM (%) | 26.3 ± 5.2 | 24.9 ± 4.0 | 0.019 |

BW, body weight; BMI, body mass index; WC, waist circumference; HC, hip circumference; WHR, waist-to-hip ratio; LBM, lean body mass; FM, fat mass; BF%, body fat percentage; PerF, peripheral fat; AbdF, abdominal fat; AbdF/FM, abdominal-fat-to-total-fat-mass ratio. Data are expressed as mean ± SD.
