# Supplementary material for: The influence of body composition and fat distribution on circadian blood pressure rhythm and nocturnal mean arterial pressure dipping in patients with obesity
Source: PLoS One. 2023 Jan 31;18(1):e0281151. doi: 10.1371/journal.pone.0281151 (PMC9888712; doi:10.1371/journal.pone.0281151)
Supplement: S3 Table — (DOCX) [file pone.0281151.s005.docx]

S3 Table. Anthropometric parameters and body composition in hypertensive and normotensive men with obesity

|  | Hypertensive men (n=112) | Normotensive men (n=18) | p value |
| --- | --- | --- | --- |
| BW (kg) | 133.7 ± 22.0 | 126.9 ± 15.5 | 0.209 |
| BMI (kg/m^2^) | 42.4 ± 6.6 | 40.5 ± 5.0 | 0.244 |
| WC (cm) | 133 ± 18 | 126 ± 12 | 0.197 |
| HC (cm) | 125 ± 15 | 119 ± 12 | 0.218 |
| WHR (cm/cm) | 1.07 ± 0.07 | 1.06 ± 0.06 | 0.837 |
| LBM (kg) | 77.6 ± 9.4 | 76.0 ± 5.9 | 0.530 |
| FM (kg) | 44.9 ± 12.4 | 47.3 ± 12.3 | 0.496 |
| BF% | 35.2 ± 5.7 | 36.9 ± 6.4 | 0.298 |
| PerF (kg) | 30.1 ± 10.4 | 33.1 ± 10.9 | 0.304 |
| AbdF (kg) | 14.8 ± 3.1 | 14.2 ± 2.4 | 0.448 |
| AbdF/FM (%) | 34.3 ± 6.6 | 31.2 ± 6.1 | 0.102 |

BW, body weight; BMI, body mass index; WC, waist circumference; HC, hip circumference; WHR, waist-to-hip ratio; LBM, lean body mass; FM, fat mass; BF%, body fat percentage; PerF, peripheral fat; AbdF, abdominal fat; AbdF/FM, abdominal-fat-to-total-fat-mass ratio. Data are expressed as mean ± SD.
