# Supplementary material for: The influence of body composition and fat distribution on circadian blood pressure rhythm and nocturnal mean arterial pressure dipping in patients with obesity
Source: PLoS One. 2023 Jan 31;18(1):e0281151. doi: 10.1371/journal.pone.0281151 (PMC9888712; doi:10.1371/journal.pone.0281151)
Supplement: Attachment — Submitted filename: Obesity_nocturnal_r2.pdf [file pone.0281151.s009.pdf]

Thank you again for the opportunity to review this manuscript. The authors have made substantial improvements to the paper. However, I still have one concern that should be addressed.

Page 8 lines 144-147.
“The 24-hour BP monitoring included 40 measurements: 32 daytime and 8 nighttime readings. The comparison of average daytime, average nighttime and mean diurnal SBP and DBP values shows that actual daytime-to-nighttime ratio of legible BP measurements was 3 to 1. That means that approximately 1/3 of the daytime readings were not legible.”

I appreciate the authors adding this section in. However, I believe more discussion is warranted about why this is a problem. Perhaps discussing this in the limitations could be useful. But ultimately, I think the authors needs to discuss why the ratio is important. Simply when looking at mean blood pressure the data is skewed to be more like the day time measurements compared to the night time measurements due to the increased number of measurements taken during the day.
