# Supplementary material for: The influence of body composition and fat distribution on circadian blood pressure rhythm and nocturnal mean arterial pressure dipping in patients with obesity
Source: PLoS One. 2023 Jan 31;18(1):e0281151. doi: 10.1371/journal.pone.0281151 (PMC9888712; doi:10.1371/journal.pone.0281151)
Supplement: Attachment — Submitted filename: BP_Bodycomp_Nocturnal.docx [file pone.0281151.s006.docx]

Thank you for the opportunity to review this manuscript. The authors sought to investigate the relationship between markers of body composition and the distribution of fat on nocturnal blood pressure dipping. The results suggest that those with the highest body weight and, BMI, and lean body mass, had reduced nocturnal blood pressure dipper. The manuscript is very interesting; however, improvement is required in some areas before it is ready for publication.

Specific comments.

Introduction

Line 54-55 “As the best currently available method of measuring blood pressure (BP), and its circadian profile, has been regarded 24-hour ambulatory blood pressure monitoring (ABPM).”

Please consider changing this line to read “24-hour ambulatory blood pressure monitoring (ABPM) has been regarded as the best currently available method of measuring blood pressure (BP), and its circadian profile.”

Additionally, who has made this claim please support this with a citation.

Methods

Lines 84-86 “Waist circumference was determined in a horizontal plane, midway

between the iliac crest and the costal margin, at the end of a normal expiration, using a non

stretch tape measure.”

Which organization recommends taking it this way? This is different than how the American College of Sports Medicine describes the waist measurement. Please clarify why it was measured this way. Additionally, how was the hip measurement determined.

Lines 97-99 “Systolic and diastolic BP were measured every 30 minutes at daytime (between 6.00 and 22.00) and every 60 minutes at night (from 22.00 to 6.00). Average daytime and nighttime BP were computed as the means of all readings during each period.”

How was mean blood pressure determined? There are 32 daytime measurements and 8 nighttime measurements. This may be problematic as this unfairly weights the influence of day time blood pressure. Honestly, I am not sure the best way to do this. But the current method is problematic. Either way please clarify how you calculated mean blood pressure.

Results.

Lines 184-185 “The assessment of nocturnal BP decline revealed that the magnitude of DBP dipping (10.8 +/- 8.7%) was significantly greater than SBP decline (7.6 +/- 6.8%, p<0.001).”

How was this determined? It is also more informative to the reader if you put the difference

Between SBP and DBP. Please clarify the percentages listed. It was stated in the statistical analyses section that it is standard deviations. That does not seem to be wha tis presented here.

Discussion

Lines 300-301 “It should be noted, that abdominal fat determined in our study comprised almost whole abdominal cavity and constituted 27,8% of”

That comma should be a period.
