# Supplementary material for: The influence of body composition and fat distribution on circadian blood pressure rhythm and nocturnal mean arterial pressure dipping in patients with obesity
Source: PLoS One. 2023 Jan 31;18(1):e0281151. doi: 10.1371/journal.pone.0281151 (PMC9888712; doi:10.1371/journal.pone.0281151)
Supplement: Attachment — Submitted filename: 5 Response to reviewers.docx [file pone.0281151.s007.docx]

Response to reviewers

Reviewer #1:

**General Comments:**

This paper explores an interesting question, however, some significant rewriting of the article for clarity and correction of grammatical error is required.

The whole article has been changed and corrected.

It would’ve been interesting to explore further how hypertension status within BMI group relates to the nocturnal blood pressure dipping status (even though non-dipping is an independent predictor of CV risk and mortality, as stated by the authors.)

As the number of normotensive patients in the study is low, the additional analysis is shown as supporting data.

**Introduction**

The rationale built here is difficult to follow. Particularly, the section on central blood pressure does not fit in. This paper does not measure central blood pressure.

The introduction has been extended and corrected to make the rationale clearer and easy to follow.

**Results**

-only report results to the decimal to which they were measured (rules of significant figures)

The results have been corrected according to the suggestion.

-line 136 and 137 – “blood pressure lowering drugs, 1-6” To what is this referring?

The sentence has been changed to make it clearer.

-Tables 2 and 6 – the “(No)” is confusing; please find a way to present this differently

‘No’ was replaced by ‘n’.

-Table 3 - as men and women should statistically significant differences in systolic and diastolic pressure that day, night, and in the mean value, it would strengthen the paper to provide further analysis examining variables with men alone and with women alone. It is interesting to note that the females alone would not even reach the hypertension classification while the males would. If this kind of analysis were added, in my opinion, some of the other analyses performed could be eliminated so as not to bloat the paper. Keep the analyses simple and related to the main purpose of the paper.

Extended analysis has been included in the supporting information files.

**Discussion**

-Line 311 – This is interesting: “whereas normotensive individuals had larger FM, BF%, and PerF.” the authors mention this finding in passing (line 250, “a possible protective role for peripheral adipose tissue” but it is not really discussed.

-line 293 - if central blood pressure measurement is arguably better, and “nocturnal SPB decline of the aorta and central arteries was significantly less pronounced compared with brachial SBP” (refs 42,43), how important are the peripheral nocturnal blood pressure measurements? What was the relationship between central dipping and peripheral dipping in other studies?

The discussion on the role of subcutaneous fat has been expanded significantly.
The role of peripheral nocturnal blood pressure measurements, and the relationship between central dipping and peripheral dipping, are additionally discussed.

**Reviewer #2**

**Introduction**

Line 54-55 “As the best currently available method of measuring blood pressure (BP), and its circadian profile, has been regarded 24-hour ambulatory blood pressure monitoring (ABPM).”

Please consider changing this line to read “24-hour ambulatory blood pressure monitoring (ABPM) has been regarded as the best currently available method of measuring blood pressure (BP), and its circadian profile.”

Additionally, who has made this claim please support this with a citation.

The sentence has been changed according to the suggestion. A citation has been added.

**Methods**

Lines 84-86 “Waist circumference was determined in a horizontal plane, midway between the iliac crest and the costal margin, at the end of a normal expiration, using a non stretch tape measure.”

Which organization recommends taking it this way? This is different than how the American College of Sports Medicine describes the waist measurement. Please clarify why it was measured this way. Additionally, how was the hip measurement determined.

There are different waist circumference measurement protocols, and there is no consensus.

The measurement ‘midway between the iliac crest and the costal margin’ is recommended by the WHO, ASTM International and ISO 7250-1:2017, among others. By taking the measurement ‘midway between the iliac crest and the costal margin’, we wanted to obtain better reliability due to stable landmarks and better correlation with visceral adipose tissue mass (the abdomen in people with obesity is more in the shape of a ball than an hour-glass).

Hip circumference was measured at the level of the femoral great trochanter.

Piqueras P et al. Anthropometric indicators as a tool for diagnosis of obesity and other health risk factors: a literature review. Front Psychol 2021;12:631179. doi: 10.3389/fpsyg.2021.631179

Serviente C, Sforzo GA. A simple yet complicated tool measuring waist circumference to determine cardiometabolic risk. ACSM’s Health & Fitness Journal [2013; 17: 29-34](https://journals.lww.com/acsm-healthfitness/toc/2013/11000).

doi: 10.1249/FIT.0b013e3182a956f5

Lines 97-99 “Systolic and diastolic BP were measured every 30 minutes at daytime (between 6.00 and 22.00) and every 60 minutes at night (from 22.00 to 6.00). Average daytime and nighttime BP were computed as the means of all readings during each period.”

How was mean blood pressure determined? There are 32 daytime measurements and 8 nighttime measurements. This may be problematic as this unfairly weights the influence of day time blood pressure. Honestly, I am not sure the best way to do this. But the current method is problematic. Either way please clarify how you calculated mean blood pressure.

I have to agree that ‘this unfairly weights the influence of daytime blood pressure’. The mean 24-hour values of SBP and DBP presented in the paper were 127.8 and 75.4 mmHg, respectively. Assuming that all 40 measurements (32 + 8) are legible, mean SBP and DBP should be 128.5 and 75.9 mmHg, respectively (daytime/nighttime measurements in the proportion of 4 to 1). If we calculate mean 24-hour SBP and DBP values including diurnal and nocturnal measurements in a 2-to-1 ratio (16 + 8 hours), the average SBP and DBP should be 127.2 and 74.8 mmHg, respectively. This means that approximately 1/3 of the daytime readings were not legible, and that the real daytime-to-nighttime measurement ratio was approximately 3 to 1.

Unfortunately, the exact numbers of legible measurements were not collected in our database, and are contained in individual patients’ source data only.

In the paper we decided to write that ‘the average circadian systolic and diastolic BP were calculated as the means of all legible measurements within a 24-hour period.’

**Results.**

Lines 184-185 “The assessment of nocturnal BP decline revealed that the magnitude of DBP dipping (10.8 +/- 8.7%) was significantly greater than SBP decline (7.6 +/- 6.8%, p<0.001).”

How was this determined? It is also more informative to the reader if you put the difference

Between SBP and DBP. Please clarify the percentages listed. It was stated in the statistical analyses section that it is standard deviations. That does not seem to be what is presented here.

The assessment of nocturnal BP decline revealed that the magnitude of SBP dipping (9.9 ± 8.2 mmHg) was significantly greater than DBP decline (8.5 ± 7.0 mmHg, p<0.001), while the percentage decline of DBP (10.8 ± 8.7%) was greater than SBP decline (7.6 ± 6.8%, p<0.001).

**Discussion**

Lines 300-301 “It should be noted, that abdominal fat determined in our study comprised almost whole abdominal cavity and constituted 27,8% of”. That comma should be a period.

The proper correction was made
