# Supplementary material for: The influence of body composition and fat distribution on circadian blood pressure rhythm and nocturnal mean arterial pressure dipping in patients with obesity
Source: PLoS One. 2023 Jan 31;18(1):e0281151. doi: 10.1371/journal.pone.0281151 (PMC9888712; doi:10.1371/journal.pone.0281151)
Supplement: Attachment — Submitted filename: Response to reviewers.docx [file pone.0281151.s008.docx]

**Response to reviewers**

**Reviewer #1:**

**Abstract**
• Still some minor grammatical correct / punctuation issues
**Intro**
• Significantly improved! - although still some minor grammatical correct / punctuation issues
**Discussion**
The grammar and punctuation issues have clearly been addressed although some minor grammar and punctuation issues remain. Review your comma usage particularly (while comma usage can be subjective, there are a few cases throughout the paper where the usage is clearly incorrect. For example, “Previous studies 288 have documented, that mean arterial pressure better reflected central, aortic SBP, and better 289 predicted cardiovascular risk, as compared with brachial SBP [38,39].” Between “documented” and “that” there clearly should not be a comma. This type of error occurs in other places.

The text has been corrected. We believe no more grammar and punctuation issues remained.

**Results**
• decimals are still shown for values for which it was unlikely the original measurement was to a decimal (age and BP, for example)

Decimals were removed. The only exception can be found on page 12, lines 198-199, as the differences between mean BP values are very small.

• “Blood pressure-lowering drugs, 1 137 through 6, have been taken by 289 participants” where can the reader find the 1-6 info?

The sentence has been changed to make it more clear (lines 150-151).

**Reviewer #2:**

Thank you for the opportunity to re-review this manuscript. The authors addressed most of my comments. I have still have three points that need to be addressed.

Page 5 lines 90-94
“Waist and hip circumferences (WC, HC) were measured to the nearest 1 cm, and the waist-to-hip ratio (WHR) was calculated. WC was determined in a horizontal plane, midway between the iliac crest and the costal margin, at the end of a normal expiration; HC was measured at the level of the greater trochanter, using a non-stretch tape measure.”
Sorry if my last comment on this was not clear. I agree that there is no consensus on how to measure waist circumference. I am just suggesting that the authors provide a reference for who recommends this method of measurement.

A reference has been provided. Line 94 [19].

Page 6 lines 105-112
“Systolic and diastolic BP (DBP) were measured every 30 minutes during the day (between 6.00 and 22.00) and every 60 minutes during the night (from 22.00 to 6.00). The average daytime and nighttime BP were computed as the means of all readings during each period, and the average circadian SBP and DBP were calculated as the means of all legible measurements within a 24-hour period. Hypertension was diagnosed according to the 2018 European Society of Cardiology guidelines [7]. Non-dipping of BP was defined as a decline in MAP values <10% from the average daytime to the average nighttime values.
“I have to agree that ‘this unfairly weights the influence of daytime blood pressure’. The mean 24-hour values of SBP and DBP presented in the paper were 127.8 and 75.4 mmHg, respectively. Assuming that all 40 measurements (32 + 8) are legible, mean SBP and DBP should be 128.5 and 75.9 mmHg, respectively (daytime/nighttime measurements in the proportion of 4 to 1). If we calculate mean 24-hour SBP and DBP values including diurnal and nocturnal measurements in a 2-to-1 ratio (16 + 8 hours), the average SBP and DBP should be 127.2 and 74.8 mmHg, respectively. This means that approximately 1/3 of the daytime readings were not legible, and that the real daytime-to-nighttime measurement ratio was approximately 3 to 1.
Unfortunately, the exact numbers of legible measurements were not collected in our database, and are contained in individual patients’ source data only.
In the paper we decided to write that ‘the average circadian systolic and diastolic BP were calculated as the means of all legible measurements within a 24-hour period.”
I appreciate the authors honesty. But they need to explicitly state this in the manuscript. It is important the reader knows how the data was affected by the unequal number of measurements.

The extended information on BP measurements was added: page 8, lines 144-147.

Page 12 lines 194-198
The assessment of nocturnal BP decline revealed that the magnitude of SBP dipping (9.9 ± 8.2 mmHg) was significantly greater than that of the DBP dipping (8.5 ± 7.0 mmHg, p<0.001), while the percentage of the DBP decline (10.8 ± 8.7%) was greater than that of SBP decline (7.6 ± 6.8%, p<0.001). The average MAP dipping was 9.4 ± 7.5%. Reduced nocturnal MAP decline was diagnosed in 219 patients (50.2%).
The authors should consider reported the difference and SD of that difference between the SBP and DBP dipping. That is the information we are interested in.

The data on the difference and SD of that difference between the SBP and DBP dipping has been added: page 12, lines 198-201.
